# Enzymatically Hydrolyzed Eggshell Membrane Promotes Keratinocyte Differentiation Associated With PKC and Akt Signaling Pathways

**DOI:** 10.1111/1750-3841.71412

**Published:** 2026-08-26

**Authors:** Kyohei Furukawa, Chika Ando, Tetsuro Kataoka, Yukio Hasebe, Hisanori Kato, Huijuan Jia

**Affiliations:** ^1^ Graduate School of Agricultural and Life Sciences The University of Tokyo Tokyo Japan; ^2^ Animal Nutrition, Graduate School of Bioagricultural Sciences Nagoya University Nagoya Japan; ^3^ ALMADO Inc. Tokyo Japan; ^4^ Nutritional Biochemistry, Department of Applied Nutrition Undergraduate School of Nutrition Sciences Japan Nutrition University Sakado Japan

## Abstract

Skin aging is characterized by physiological and morphological changes, and nutritional interventions are key strategies to mitigate this process. Eggshell membrane (ESM), a natural byproduct of egg processing, is typically discarded as industrial waste; however, recent studies have revealed its health benefits, including antiobesity and anti‐inflammatory effects. We previously reported that enzymatically hydrolyzed ESM (eESM) promotes keratinocyte differentiation in NHEK‐Neo cells, although the underlying mechanisms remain unclear. In this study, we show that treatment of NHEK‐Neo cells with varying concentrations of eESM upregulated keratinocyte differentiation markers (*KRT1*, *KRT10*, *IVL*, *FLG*, *KRT13*) and induced dose‐dependent morphological changes. Calcium, a key regulator of keratinocyte differentiation, is present in eESM. Supplementation of calcium significantly increased transcription of early differentiation markers (*KRT1*, *KRT10*) but had little effect on late markers (*IVL*, *FLG*, *KRT13*), suggesting that additional bioactive components in eESM contribute to differentiation. Furthermore, eESM enhanced the expression of PKC‐related genes (*IRF6*, *GRHL3*, *OVOL1*) and increased phosphorylation of Akt and ERK2. Pharmacological inhibition of PKC and Akt signaling suppressed keratinocyte differentiation. Ultra‐high‐performance liquid chromatography analysis further suggested that eESM may contain components with a molecular weight of approximately 1000 Da, although further characterization is required. Collectively, these findings indicate that eESM promotes keratinocyte differentiation associated with PKC and Akt signaling pathways, likely through the coordinated action of calcium and other bioactive molecules, and that the observed effects cannot be attributed to calcium alone. eESM may, therefore, represent a potential food‐derived functional ingredient for skin health.

## 1 Introduction

The aging population is rapidly increasing in developed countries, and the global population aged over 60 is projected to rise by 22% by 2050 (Rahimi et al. 2014). Aging involves time‐dependent changes in cellular metabolism, physiology, and function, which are associated with increased disease morbidity and mortality (Catic 2018; Höhn et al. 2017; Rae et al. 2010), as well as a decline in health‐related quality of life. The skin, the largest organ in the human body, plays various essential roles, including protection, thermoregulation, and sensory perception. Human skin is continuously exposed to both internal and external stimuli, leading to the progression of skin aging, which manifests as dryness, wrinkling, hair graying, scalp hair thinning or baldness, and thinning of the skin. Skin aging is generally categorized into two types: chronological aging and photoaging (Tobin 2017). Chronological aging is driven by internal factors such as hormonal changes and genetic predisposition, which naturally progress with age and are difficult to regulate. In contrast, photoaging is caused by external factors including ultraviolet radiation, nutritional status, and chemical pollutants. Therefore, photoaging can potentially be delayed through interventions such as balanced nutrition, functional bioactive compounds, and skincare products (Calvo et al. 2024; Cao et al. 2020; Csekes and Račková 2021; Mukherjee et al. 2011).

Eggshell membrane (ESM) constitutes approximately 1.02% of the egg structure (Han et al. 2023). It is a natural byproduct of egg processing and is typically discarded as industrial waste, contributing to environmental burden. Nutritionally, ESM is composed of approximately 90% protein, 3% lipids, 2% carbohydrates, and small amounts of minerals such as calcium and magnesium (Han et al. 2023). Although limited information is available on the specific proteins in ESM due to its insolubility and protein instability, it is known to be rich in fibrous proteins, collagen, hyaluronic acid, chondroitin sulfate, and glucosamine (Ruff et al. 2009), suggesting its potential utility in health science. Previous studies have demonstrated that powdered ESM (pESM) exhibits antiobesity, anti‐inflammatory, and antiaging effects in rodent models (Jia et al. 2017; Jia et al. 2022; Ramli et al. 2020; Warman et al. 2024; Yang et al. 2022). Moreover, our previous research showed that enzymatically hydrolyzed ESM (eESM) promotes keratinocyte differentiation and restores skin aging phenotypes, such as skin thickness, in interleukin‐10 knockout mice (Furukawa et al. 2021). These findings suggest that ESM contains bioactive compounds capable of mitigating aging‐related cellular dysfunction in the skin.

The epidermis, the outermost layer of the skin, is the site of keratinocyte differentiation. In the epidermis, keratinocyte proliferation is confined to the basal layer, while differentiation occurs as cells migrate to the spinous and granular layers. A key trigger for keratinocyte differentiation is an increase in extracellular calcium levels (Elsholz et al. 2014). Calcium influx into keratinocytes promotes the transcription of differentiation markers such as keratins (KRT1, KRT10, KRT13), involucrin (IVL), filaggrin (FLG), and transglutaminase 1 (TGM1). This calcium‐induced differentiation is regulated by complex signaling mechanisms. One important molecule is protein kinase C (PKC), which is activated by calcium and interacts with receptor‐interacting protein kinase 4 (RIPK4) to promote keratinocyte differentiation (Xu et al. 2020). Protein kinase B (Akt) also plays a critical role in the transcription of KRT1, and its deletion leads to abnormal epidermal development (Thrash et al. 2006). Additionally, the Notch signaling pathway is essential for keratinocyte growth arrest and initiation of differentiation (Rangarajan et al. 2001).

Given the potential biological activities of ESM and its capacity to promote keratinocyte differentiation, the molecular mechanism underlying its effects remains to be elucidated. Therefore, in the present study, we investigated the underlying mechanisms of eESM‐induced differentiation in neonatal normal human epidermal keratinocytes (NHEK‐Neo). In addition, we explored the molecular properties of the bioactive compounds within eESM in comparison with other ESM preparations, including pESM and sodium hydroxide‐hydrolyzed ESM (hESM). While our previous study demonstrated the beneficial effects of ESM on keratinocyte differentiation (Furukawa et al. 2021), the molecular mechanisms underlying these effects have not been fully elucidated. In particular, the present study provides new insights into the involvement of TRPV3‐mediated calcium signaling and downstream PKC and PI3K–Akt pathways.

## 2 Materials and Methods

### 2.1 Cell Culture

Neonatal Normal Human Epidermal Keratinocytes (NHEK‐Neo) were purchased from Lonza Japan (Tokyo, Japan) and cultured in KGM‐Gold Keratinocyte Growth Medium BulletKit (#00192060, Lonza) consisting of KBM‐Gold Basal Medium and KGM‐Gold SingleQuots supplements. Cells were maintained at 37°C in a humidified atmosphere containing 5% CO_2_. eESM and hESM were prepared from pESM by papain and sodium hydroxide treatment, respectively, as previously described (Furukawa et al. 2021). eESM was added to the culture medium at final concentrations ranging from 0.5 to 4.0 mg/mL. To assess the effect of extracellular calcium, cells were treated with different concentrations of calcium chloride. Morphological changes were monitored using a microscope (Leica Microsystems GmbH, Wetzlar, Germany).

For inhibitor experiments, the PKC inhibitor GF109203X and the phosphatidylinositol 3‐kinase inhibitor LY294002 (both from Abcam, Cambridge, UK) were added to the culture at final concentrations of 5 or 10 µM in combination with eESM.

### 2.2 RNA Extraction From NHEK‐Neo Cells

Total RNA was extracted using the Nucleospin RNA Plus Kit (Takara Bio Inc., Shiga, Japan), as previously described (Furukawa et al. 2021; Furukawa et al. 2022). RNA concentration and purity were assessed using a NanoDrop ND‐1000 spectrophotometer (Thermo Scientific, Waltham, MA, USA). Complementary DNA (cDNA) was synthesized using PrimeScript RT Master Mix (Takara Bio Inc.) according to the manufacturer's instructions. Real‐time RT‐PCR was performed using SYBR Premix Ex Taq (Takara Bio Inc.) and the Thermal Cycler Dice Real Time System TP800 (Takara Bio Inc.). Primer sequences are listed in Table S1. Gene expression levels were normalized to B2M and expressed as fold changes relative to the control group.

### 2.3 Measurement of Calcium Concentrations

Calcium concentrations in the culture medium were measured using the Amplite Fluorimetric Calcium Quantitation Kit (AAT Bioquest, Pleasanton, CA, USA) following the manufacturer's protocol. Equal volumes of medium and working solution were mixed and incubated for 20 min, protected from light. Fluorescence was measured using a VARIOSCAN LUX microplate reader (Thermo Scientific).

### 2.4 Reanalysis of DNA Microarray Data

Upstream analysis was performed using Ingenuity Pathway Analysis (IPA, http://www.ingenuity.com/, accessed on September 29, 2020). The dataset used was from our previous study comparing NHEK‐Neo cells treated with 0 or 1.0 mg/mL eESM for 24 h (Furukawa et al. 2021), available in the GEO database under accession number GSE178597. Genes with absolute activation Z‐score > 1 were selected, and PKC, Akt, and Notch signaling pathways were examined.

### 2.5 Western Blotting Analysis

Cells were lysed in radioimmunoprecipitation assay (RIPA) buffer (Cell Signaling Technology, Danvers, MA, USA), and protein concentrations were determined using the Pierce BCA Protein Assay Kit (Thermo Scientific). Protein samples (1.8 µg/µL) were mixed with 3× Laemmli sample buffer containing 189 mM Tris‐HCl (pH 6.8), 6% SDS, 30% glycerol, 0.0045% bromophenol blue, and 15% 2‐mercaptoethanol, then heated at 95°C for 5 min. Equal amounts of protein (12 µg per lane) were separated by SDS‐PAGE using 8% polyacrylamide gels and transferred to polyvinylidene difluoride (PVDF) membranes (Immobilon‐P, Merck Millipore, Burlington, MA, USA) using a Mini Trans‐Blot Cell (Bio‐Rad, Hercules, CA, USA).

Membranes were blocked with PVDF Blocking Reagent for Can Get Signal (TOYOBO, Osaka, Japan). Primary and secondary antibodies were diluted using Can Get Signal Immunoreaction Enhancer Solutions I and II (TOYOBO), respectively. Signals were detected using ECL Prime Western Blotting Detection Reagent (GE Healthcare, Chicago, IL, USA) and visualized with Ez‐CaptureMG (ATTO, Tokyo, Japan). Antibody details are provided in Table S2.

### 2.6 Size Exclusion Chromatography

To estimate the molecular mass of bioactive compounds in eESM, ultra‐high‐performance liquid chromatography (UHPLC) was performed using three types of ESM: eESM, pESM, and hESM. Samples were dissolved in 0.1 M potassium phosphate buffer (pH 7.0) and filtered through a 0.22 µm PVDF membrane. Mass calibration standards (ProteoMass Peptide and Protein MALDI‐MS Calibration Kit, Supelco, Bellefonte, PA, USA) were dissolved in 0.1% trifluoroacetic acid (TFA) or a 50:50 mixture of TFA and acetonitrile.

Samples (20 µL) were injected into a Nexera X2 UHPLC system (Shimadzu, Kyoto, Japan) equipped with a Shim‐pack Bio Diol‐60 column (4.6 mm i.d. × 300 mm, 3.0 µm; Shimadzu GLC, Tokyo, Japan). The mobile phase consisted of 0.1 M potassium phosphate buffer (pH 7.0) containing 0.2 M sodium chloride, with a flow rate of 0.5 mL/min. The column was maintained at 25°C, and absorbance was monitored at 215 nm.

### 2.7 Statistical Analysis

Data are presented as mean ± standard error. Statistical significance was determined using Tukey–Kramer's multiple comparison test. Correlation analysis was performed using Pearson's correlation test. A *p*‐value < 0.05 was considered statistically significant.

## 3 Results and Discussion

### 3.1 Effects of Different Concentrations of eESM on Keratinocyte Differentiation

**FIGURE 1:**
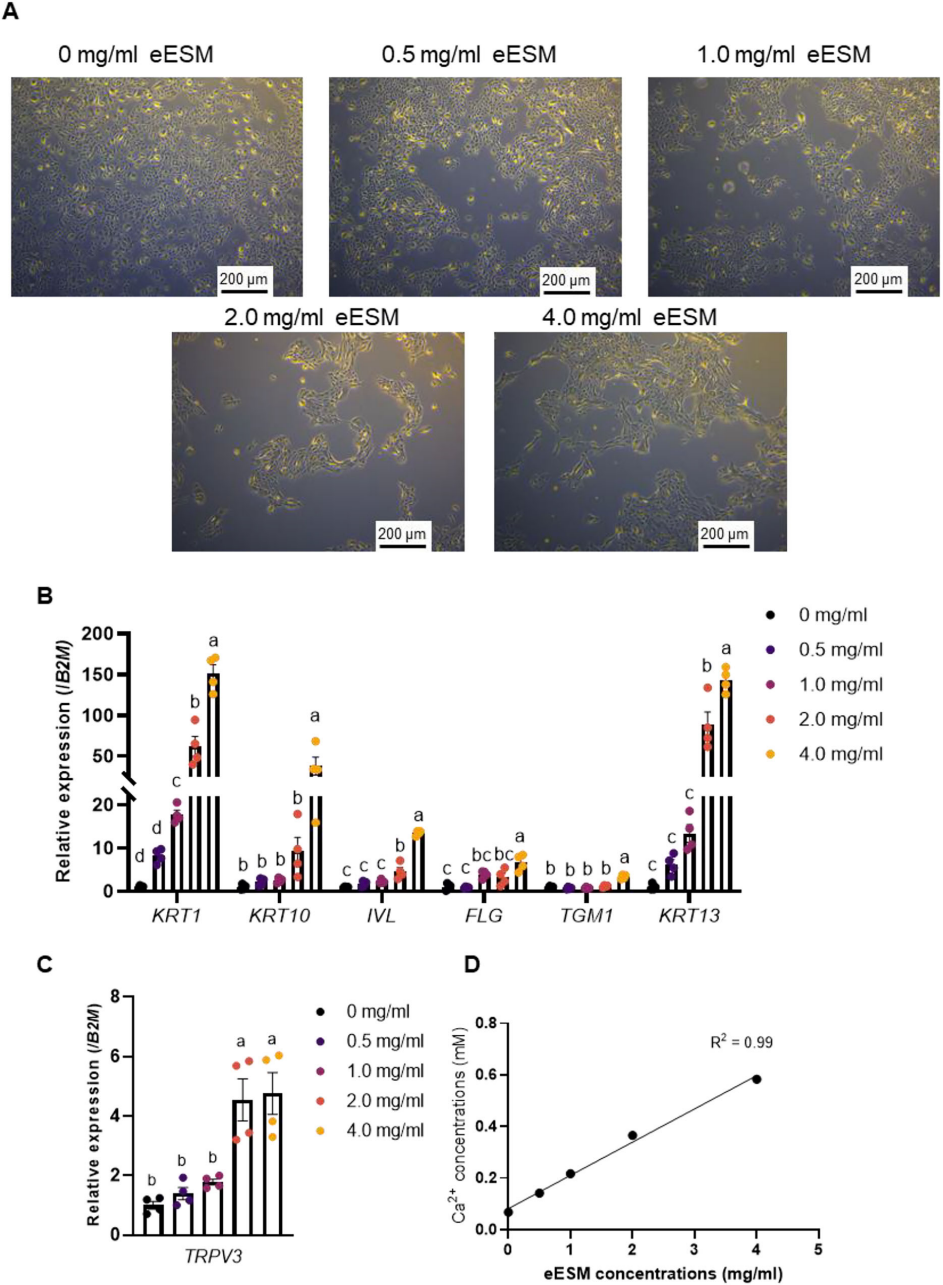
Effects of different concentrations of eESM treatment on keratinocyte differentiation and calcium ion uptake. (A) NHEK‐Neo cells were treated with 0.5−4.0 mg/mL eESM for 4 days. Morphological changes were observed by a microscope. The scale bar shows 200 µm. (B, C) After eESM treatment, the expression of keratinocyte differentiation‐related gene and TRPV3 were investigated by real‐time RT‐PCR. (D) Calcium ion concentration in eESM‐contained medium was examined. Data were expressed as mean ± SE (*n* = 4 biological replicates). Statistical analysis was performed with Tukey−Kramer's multiple comparison test. The different letters (a, b, c, d) on the graph indicate statistical significance (*p* < 0.05).

Cell morphology changed to a flattened appearance after 5 days of treatment with 1.0 mg/mL eESM (Figure 1A), consistent with our previous findings (Furukawa et al. 2021). These morphological changes became more pronounced with increasing eESM concentrations, indicating a dose‐dependent response.

Gene expression analysis of keratinocytes differentiation markers is shown in Figure 1B. eESM treatment significantly upregulated the expression of *KRT1*, *KRT10*, and *KRT13* in a dose‐dependent manner. Although the changes were less pronounced, *IVL*, *FLG*, and *TGM1* expression also increased following eESM treatment. Given that KRT1 and KRT10 are early‐stage differentiation markers, and KRT13, IVL, and FLG are late‐stage markers, these results suggest that eESM promotes keratinocyte differentiation at both morphological and gene expression levels.

TRPV3 is a calcium‐permeable cation channel expressed in keratinocytes and is implicated in multiple epidermal functions, including differentiation, barrier formation, and tissue regeneration (Aijima et al. 2015; Cheng et al. 2010; Guo et al. 2023; He et al. 2015; Wang et al. 2021; Zhang et al. 2018). Our previous study showed that *TRPV3* expression was increased by 1.0 mg/mL eESM (Furukawa et al. 2021). This trend was confirmed in the current dose‐dependent experiment, with significant increases observed at 2.0 and 4.0 mg/mL eESM (Figure 1C), suggesting that TRPV3‐mediated calcium signaling contributes to eESM‐induced keratinocyte differentiation.

### 3.2 Effects of Calcium on Keratinocyte Differentiation

Although few studies have examined calcium‐induced differentiation in NHEK‐Neo cells, extracellular calcium is a well‐established trigger of differentiation in HaCaT cells and mouse primary keratinocytes (Bikle et al. 2012; Boukamp et al. 1988; Elsholz et al. 2014; Palazzo et al. 2017). Because the mineralized layer of ESM contains high levels of calcium carbonate (Han et al. 2023), we next examined whether calcium derived from eESM contributes to keratinocyte differentiation.

**FIGURE 2:**
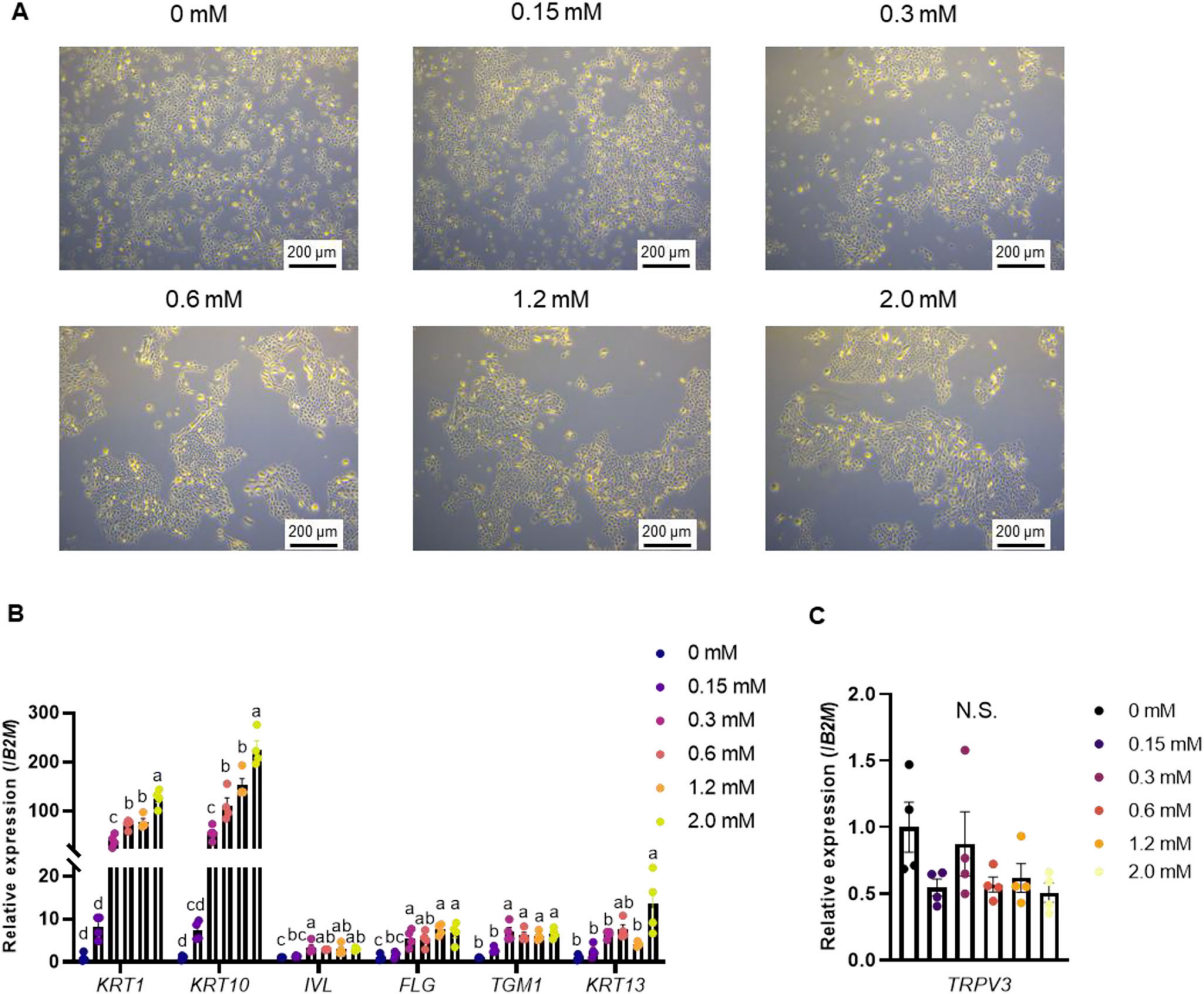
Effects of different concentrations of calcium treatment on keratinocyte differentiation and calcium ion uptake. (A) NHEK‐Neo cells were treated with 0.15−2.0 mM calcium for 4 days. Morphological changes were observed by a microscope. The scale bar shows 200 µm. (B, C) After treatment, the expression of keratinocyte differentiation‐related gene and TRPV3 were investigated by real‐time RT‐PCR. Data were expressed as mean ± SE (*n* = 4 biological replicates). Statistical analysis was performed with Tukey−Kramer's multiple comparison test. The different letters (a, b, c, d) on the graph indicate statistical significance (*p* < 0.05).

As shown in Figure 1D, calcium ion concentrations in the medium increased with increasing eESM concentrations. To test whether calcium ions derived from eESM contribute to keratinocyte differentiation, NHEK‐Neo cells were treated with various concentrations of calcium chloride. Morphological analysis revealed that calcium‐treated cells exhibited aggregation compared to untreated controls (Figure 2A). Early differentiation markers *KRT1* and *KRT10* were significantly upregulated in a dose‐dependent manner (Figure 2B), confirming its role as an initiator of keratinocyte differentiation. In contrast, late differentiation markers *IVL*, *FLG*, and *KRT13* showed only modest increases, which were less pronounced than those observed with eESM treatment. In addition, *TRPV3* expression remained unchanged across all calcium concentrations (Figure 2C). These findings indicate that while calcium serves as a key initiator of NHEK‐Neo differentiation, the differentiation induced by eESM cannot be fully explained by calcium alone. Notably, the calcium concentration achieved with 4.0 mg/mL eESM (∼0.8 mM) is lower than the levels typically required (1.5–2.0 mM) to fully induce keratinocyte differentiation, suggesting that additional bioactive components in eESM contribute to the observed effects.

### 3.3 Signaling Pathways Involved in eESM‐Induced NHEK‐Neo Differentiation

Based on previous evidence that TRPV3‐mediated calcium signaling can activate EGFR–PI3K/Akt pathways and calcium‐dependent PKC signaling in keratinocytes (Aijima et al. 2015; Guo et al. 2023; Palazzo et al. 2017), we reanalyzed DNA microarray data from our previous study, which compared NHEK‐Neo cells treated with 0 or 1.0 mg/mL eESM. Upstream analysis revealed activation of PKC (Z‐score: 2.017) and suppression of Akt (Z‐score: −1.197). Although Notch signaling has been implicated in keratinocyte differentiation, it was not identified as a predominant pathway under the present experimental conditions. Gene lists associated with PKC and Akt pathways are shown in Tables 1 and 2.

**TABLE 1:** Results of upstream analysis on PKC signaling using previous microarray analysis.

Gene symbol	Gene name	Fold change (eESM vs. control)
Activating Z‐score: 2.017		
*ABCA1*	ATP binding cassette subfamily A member 1	3.87
*ADM*	Adrenomedullin	3.2
*CDC6*	Cell division cycle 6	−12.5
*CDKN1A*	Cyclin‐dependent kinase inhibitor 1A	2.1
*CPT1A*	Carnitine palmitoyltransferase 1A	2.34
*CXCL8*	Chemokine (C‐X‐C motif) ligand 8	−5.71
*DDIT3*	DNA‐damage‐inducible transcript 3	3.4
*EGR1*	Early growth response 1	3.28
*FGF2*	Fibroblast growth factor 2	2.76
*FGFBP1*	Fibroblast growth factor binding protein 1	−3.2
*FN1*	Fibronectin 1	6.75
*GADD45A*	Growth arrest and DNA‐damage‐inducible, alpha	2.13
*GRHL3*	Grainyhead‐like transcription factor 3	16.75
*HMGCR*	3‐hydroxy‐3‐methylglutaryl‐coa reductase	2.84
*HMOX1*	Heme oxygenase 1	7.1
*HPGD*	Hydroxyprostaglandin dehydrogenase 15‐(NAD)	3.62
*IL6*	Interleukin 6	−7.77
*IRF6*	Interferon regulatory factor 6	3.93
*IVL*	Involucrin	27.13
*JUN*	Jun proto‐oncogene	−3.99
*MMP1*	Matrix metallopeptidase 1	4.98
*MMP2*	Matrix metallopeptidase 2	−2.2
*MMP9*	Matrix metallopeptidase 9	23.11
*NR1D1*	Nuclear receptor subfamily 1, group D, member 1	3.54
*OVOL1*	Ovo‐like zinc finger 1	12.04
*PPARA*	Peroxisome proliferator‐activated receptor alpha	2.71
*RBL1*	Retinoblastoma‐like 1	−3.14
*RGS2*	Regulator of G‐protein signaling 2	2.43
*TGM1*	Transglutaminase 1	5.21

**TABLE 2:** Results of upstream analysis on Akt signaling using previous microarray analysis.

Gene symbol	Gene name	Fold change (eESM vs. control)
Activating Z‐score: −1.197		
*ADM*	Adrenomedullin	3.2
*BCL6*	B‐cell CLL/lymphoma 6	4.9
*BIRC5*	Baculoviral IAP repeat containing 5	−5.92
*CD274*	CD274 molecule	−3.7
*CDH1*	Cadherin 1, type 1	2.27
*CDKN1A*	Cyclin‐dependent kinase inhibitor 1A	2.1
*CFLAR*	CASP8 and FADD like apoptosis regulator	2.56
*CPT1A*	Carnitine palmitoyltransferase 1A	2.34
*CTSD*	Cathepsin D	4.86
*CXCL8*	Chemokine (C‐X‐C motif) ligand 8	−5.71
*ETS1*	v‐ets avian erythroblastosis virus E26 oncogene homolog 1	−2.45
*EZH2*	Enhancer of zeste 2 polycomb repressive complex 2 subunit	−2.0
*FOXO3*	Forkhead box O3	2.84
*GSR*	Glutathione reductase	−4.07
*IL6*	Interleukin 6	−7.77
*IL7R*	Interleukin 7 receptor	−12.31
*LCN2*	Lipocalin 2	10.52
*MMP2*	Matrix metallopeptidase 2	−2.2
*MMP9*	Matrix metallopeptidase 9	23.11
*MUC1*	Mucin 1, cell surface associated	6.81
*MYC*	v‐myc avian myelocytomatosis viral oncogene homolog	−2.45
*NOTCH2*	Notch 2	3.11
*PEA15*	Phosphoprotein enriched in astrocytes 15	2.46
*RRM2*	Ribonucleotide reductase M2	−48.9
*SLC4A7*	Solute carrier family 4, sodium bicarbonate cotransporter, member 7	−2.46
*TFAM*	Transcription factor A, mitochondrial	−3.17
*TNC*	Tenascin C	−6.08

**FIGURE 3:**
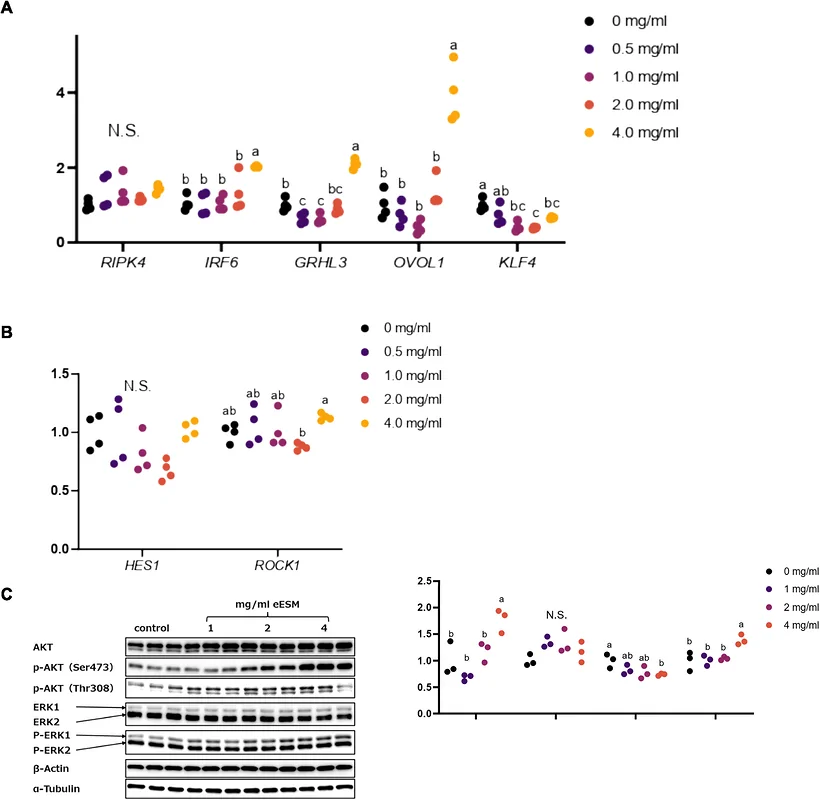
Involvement of PKC, Akt, and Notch signaling pathway in eESM‐induced keratinocyte differentiation. (A, B) The expression of PKC (A) and Notch (B) signaling pathway‐related genes were examined by real‐time RT‐PCR. Data are expressed as mean ± SE (*n* = 4 biological replicates). (C) The phosphorylation levels of Akt and ERK1/2 were investigated by western blotting. Representative blots are shown. Quantification was performed using *n* = 3 biological replicates. Statistical analysis was performed with Tukey−Kramer's multiple comparison test. The different letters (a, b, c) on the graph indicate statistical significance (*p* < 0.05).

Key PKC‐related genes, including *GRHL3* and *OVOL1*, were upregulated by 1.0 mg/mL eESM. We further examined the expression of PKC‐ and Notch‐related genes in cells treated with varying eESM concentrations. As shown in Figure 3A, *GRHL3* and *OVOL1* expressions were significantly increased at 4.0 mg/mL eESM, but not at 1 mg/mL. A similar trend was observed for *IRF6*, while *RIPK4* and *KLF4* expressions were unchanged or decreased. In the Notch pathway, *HES1* and *ROCK1* expressions remained unchanged across all eESM concentrations (Figure 3B).

As shown in Figure 3C and Figure S1, Akt phosphorylation at Ser473 significantly increased by 4.0 mg/mL eESM, contrary to the microarray data. A similar trend was observed for pERK2/t‐ERK2. These results suggest that eESM may activate PKC and Akt‐ERK signaling pathways in NHEK‐Neo cells.

**FIGURE 4:**
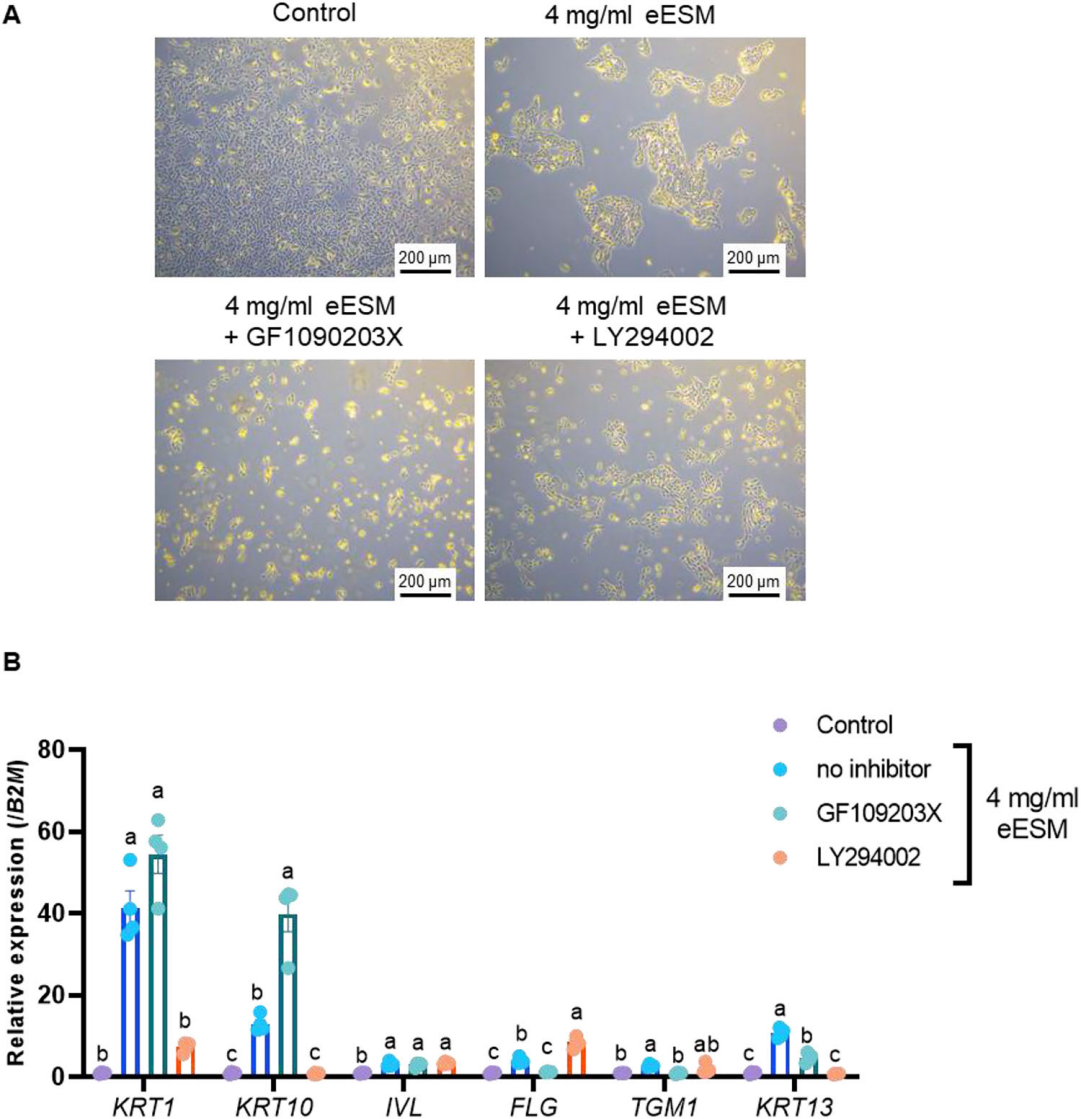
Pharmacological inhibition of Akt and PKC signaling pathway in NHEK‐Neo cells treated with 4.0 mg/mL of eESM. (A) The inhibitor of PKC (GF 109203X) and phosphatidylinositol 3‐kinase (LY 294002) were exposed to 4 mg/mL eESM‐treated NHEK‐Neo cells. Morphological changes were observed by a microscope. The scale bar shows 200 µm. (B) The expression of keratinocyte differentiation markers was investigated by real‐time RT‐PCR. Data were expressed as mean ± SE (*n* = 4 biological replicates). Statistical analysis was performed with Tukey−Kramer's multiple comparison test. The different letters (a, b, c) on the graph indicate statistical significance (*p* < 0.05).

To confirm the involvement of these pathways, we used inhibitors targeting PKC (GF109203X) and PI3K‐Akt (LY294002). These pharmacological inhibitors are widely used to modulate PKC and PI3K–Akt signaling pathways in keratinocytes and related epithelial cell systems (Shrestha et al. 2016), supporting the validity of this experimental approach. Lactate dehydrogenase assays showed no increase in cytotoxicity at 5 µM of either inhibitor in the presence of 4.0 mg/mL eESM (Figure S2). Morphological analysis revealed that the flattening effect of eESM was abolished by both inhibitors (Figure 4A).

Gene expression analysis showed that *KRT1* and *KRT10* upregulation by eESM was significantly suppressed by LY294002 but not by GF109203X (Figure 4B). In contrast, *FLG* and *TGM1* expressions were inhibited by GF109203X but not by LY294002. *KRT13* expressions were suppressed by both inhibitors, while *IVL* expressions remained unaffected. Similar trends in gene expression and cell morphology were also observed at 1.0 mg/mL eESM (Figure S3), supporting the reproducibility of eESM‐induced signaling responses. These results suggest that PI3K‐Akt signaling regulates early differentiation markers, whereas PKC signaling influences late‐stage markers in eESM‐treated NHEK‐Neo cells.

Although pharmacological inhibition provided evidence for the involvement of these pathways, further studies using genetic approaches such as siRNA‐mediated knockdown or rescue experiments will be necessary to confirm the specificity of PKC and PI3K‐Akt signaling in eESM‐induced keratinocyte differentiation.

In addition, the concentrations of eESM used in this study were selected to facilitate mechanistic analyses and may exceed physiologically achievable levels under dietary or topical exposure conditions. Further studies will be required to determine the relevance of these concentrations in vivo and to evaluate the translational potential of eESM.

### 3.4 Estimation of Molecular Size of Bioactive Molecules in eESM

To estimate the molecular size of bioactive compounds in eESM, size exclusion chromatography was performed using UHPLC. Based on our previous findings that keratinocyte differentiation was induced by eESM, but not by pESM or hESM, these three types of ESMs were compared.

**FIGURE 5:**
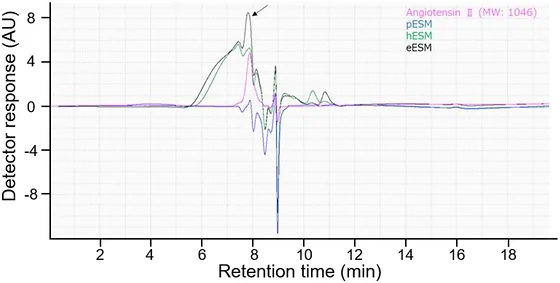
Size exclusion chromatography profiles of three ESM preparations. Powdered ESM (pESM), alkaline‐hydrolyzed ESM (hESM), and enzymatically hydrolyzed ESM (eESM) were dissolved in 0.1 M potassium phosphate buffer (pH 7.0), filtered through a 0.22 µm PVDF membrane, and analyzed by UHPLC.

As shown in Figure 5, pESM exhibited few peaks due to its high content of insoluble proteins (Kodali et al. 2011; Takahashi et al. 1996), which were likely removed during filtration. In contrast, hESM and eESM contained molecules with molecular weights ranging from 757 (Bradykinin fragment 1–7) to 16,952 (Apomyoglobin). When chromatograms were overlaid, a peak near Angiotensin II (molecular weight: 1046) was more prominent in eESM than in hESM. Although this analysis does not allow definitive molecular identification, these results suggest that eESM may contain bioactive components with a molecular weight of approximately 1000 Da.

## 4 Conclusion

**FIGURE 6:**
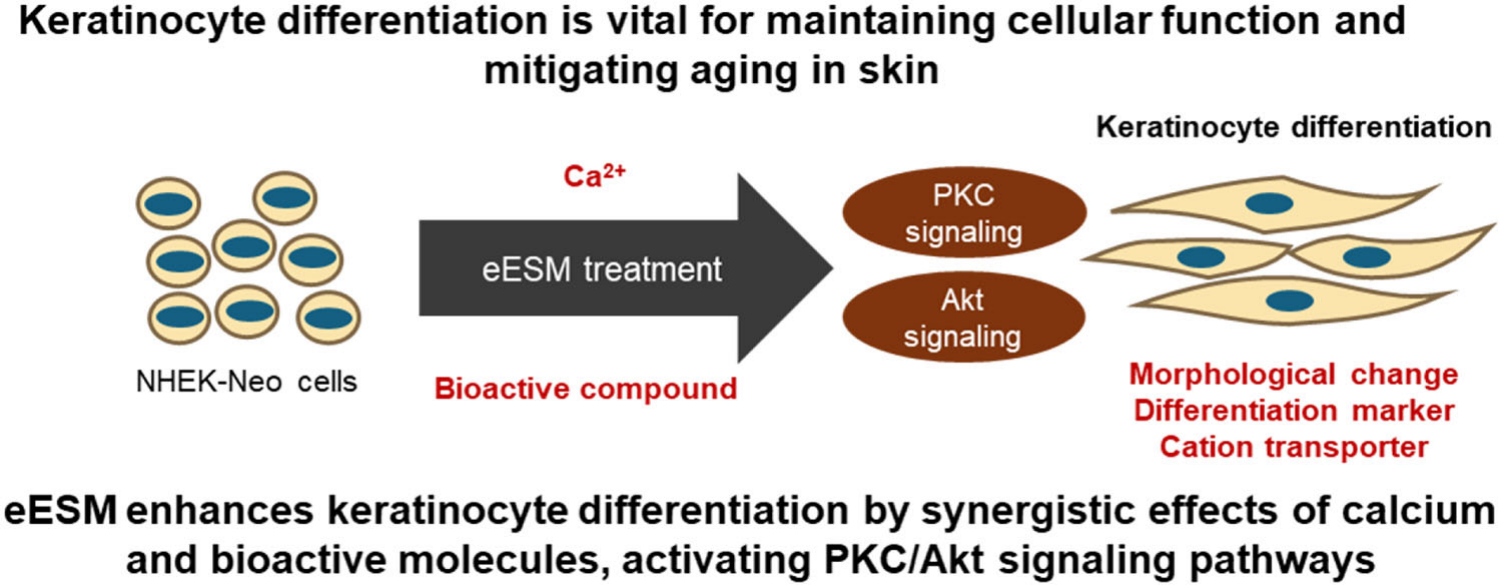
Proposed mechanism of eESM‐induced keratinocyte differentiation.

Our findings indicate that eESM contains bioactive compounds that promote keratinocyte differentiation. This process appears to involve PI3K‐Akt and PKC signaling pathways, potentially mediated by TRPV3‐dependent calcium uptake. A schematic summary of the proposed mechanism is shown in Figure 6.

Given that the ESM is a byproduct of egg production that is typically discarded as industrial waste, these findings provide insight into its potential value as a functional food–derived ingredient for skin health. Together with previous reports describing its antiobesity (Ramli et al. 2020), anti‐inflammation (Jia et al. 2017), the suppression of skin aging (Furukawa et al. 2021; Kalman and Hewlings 2020), gut dysbiosis (Yang et al. 2022), and oxidative stress (Zhu et al. 2022), eESM may represent a sustainable resource with health‑related benefits.

However, the present findings are based on in vitro experiments, and the identification of the active component was not achieved. Therefore, further in vivo studies and compound identification will be necessary to validate these effects.

## Author Contributions

**Kyohei Furukawa**: Writing – original draft, formal analysis, investigation, methodology, visualization. **Chika Ando**: Formal analysis, writing – review and editing. **Tetsuro Kataoka**: Formal analysis. **Yukio Hasebe**: Conceptualization, resources. **Hisanori Kato**: Conceptualization, supervision, project administration, funding acquisition, writing – review and editing. **Huijuan Jia**: Conceptualization, project administration, funding acquisition, writing – review and editing.

## Conflicts of Interest

The authors declare no conflicts of interest.

## Supporting information




**Supporting File 1**: jfds71412‐sup‐0001‐Table S1‐S2.docx
Table S1. Primer sequences
Table S2. Antibody information

**Supporting File 2**: jfds71412‐sup‐0002‐Figure S1‐S3.pptx
Figure S1. Uncropped western blot images corresponding to the data shown in Figure 3C. Full‐length, uncropped western blot images are shown. Boxes indicate the regions used in Figure 3C. High exposure was required to detect low‐abundance phosphorylated proteins. Molecular weight markers were not visible in chemiluminescent images and are shown separately under visible light. The membranes were cut prior to antibody incubation because of the presence of nonspecific bands; however, all samples shown were derived from the same gel.
Figure S2. Cell cytotoxicity of eESM, GF 109203X and LY294002 in NHEK‐Neo cells.
Figure S3. Effects of pharmacological inhibition of PKC and Akt signaling pathway in 1 mg/mL of eESM‐treated NHEK‐Neo cells.
