# Supplementary material for: Enzymatically Hydrolyzed Eggshell Membrane Promotes Keratinocyte Differentiation Associated With PKC and Akt Signaling Pathways
Source: J Food Sci. 2026 Aug 26;91(8):e71412. doi: 10.1111/1750-3841.71412 (PMC13507864; doi:10.1111/1750-3841.71412)
Supplement: Supplementary file 1 [file JFDS-91-0-s001.docx]

Supplemental Table 1. Primer Sequences.

| Gene | Primer sequence (5’-3’) | |
| --- | --- | --- |
| *For human* | | |
| *B2M* | *Sense* | ACACTGAATTCACCCCCACTG |
|  | *Antisense* | TGCTTACATGTCTCGATCCCAC |
| *FLG* | *Sense* | TCGGCAAATCCTGAAGAATCCA |
|  | *Antisense* | TGTGGTCTATATCCAAGTGATCCA |
| *GRHL3* | *Sense* | CCAAAGCCATCCACATCAA |
|  | *Antisense* | CGAAGACAACCATCACCACA |
| *HES1* | *Sense* | GGACATTCTGGAAATGACAGTGAA |
|  | *Antisense* | TCGGTACTTCCCCAGCACA |
| *IRF6* | *Sense* | TTGGAAAGGAAACTCATCTTGG |
|  | *Antisense* | CCTTGATGTCTGGGGTTGAG |
| *Ivl* | *Sense* | GAGTCTGGTTGACAGTAGCTT |
|  | *Antisense* | GGAGGAACAGTCTTGAGGAGC |
| *KLF4* | *Sense* | ATCTTTCTCCACGTTCGCGT |
|  | *Antisense* | CTCCCGCCAGCGGTTATTC |
| *KRT1* | *Sense* | TGGACCTTCAGGCCAAACTT |
|  | *Antisense* | TGCATCTGAGACAACTCTGCTT |
| *KRT10* | *Sense* | TCCCAACTGGCCTTGAAACA |
|  | *Antisense* | TGAGAGCTGCACACAGTAGC |
| *KRT13* | *Sense* | CAGTCATCCAGAGATGTGACCT |
|  | *Antisense* | TTGCCATCGTGCACATCCAT |
| *OVOL1* | *Sense* | AATGCAAAGGATGAGTGCAGTG |
|  | *Antisense* | TCTCTCTAGGGCAGATGAGTTC |
| *RIPK4* | *Sense* | GGATCACTGTCGCTGTCCTTT |
|  | *Antisense* | CTTCATCAGTTTGCTGGTGTCC |
| *ROCK1* | *Sense* | AACTTTTGGACCTCTCGGATTCTAC |
|  | *Antisense* | CAGCCATATCGTTTGATATTTCCTC |
| *TRPV3* | *Sense* | AACCTCACCAGCCATGAAAGC |
|  | *Antisense* | TGTGCACTCTTCTTTGTGGGG |

Supplemental Table 2. Antibody Information.

| Antibody | Company | Catalog number |
| --- | --- | --- |
| Akt | Cell Signaling Technology | 9272 |
| Phospho-Akt(Ser473) | Cell Signaling Technology | 9271 |
| Phospho-Akt(Thr308) | Cell Signaling Technology | 9275 |
| P44/42 MAPK (Erk1/2) (3A7) | Cell Signaling Technology | 3700 |
| Phospho-p44/42 MAPK (Erk1/2) (Thr202/Tyr204) (20G11) | Cell Signaling Technology | 3873 |
| β-Actin (8H10D10) | Cell Signaling Technology | 9107 |
| α-Tubulin（DM1A） | Cell Signaling Technology | 4376 |
| anti-rabbit IgG antibody | GE healthcare | NA934 |
| anti-mouse IgG antibody | GE healthcare | NA931 |
